# Supplementary material for: HIVprotI: an integrated web based platform for prediction and design of HIV proteins inhibitors
Source: J Cheminform. 2018 Mar 9;10:12. doi: 10.1186/s13321-018-0266-y (PMC5845081; doi:10.1186/s13321-018-0266-y)
Supplement: Supplementary file 2 — Additional file 2. Source code of HIVProtI web server. [file 13321_2018_266_MOESM2_ESM.zip › HIVprotI_Source-code/hivprotI/weka-3-6-6/doc/weka/classifiers/lazy/LBR.html]

LBR


---


|  |  |  |
| --- | --- | --- |
| **Overview** \| **Package** \| **Class** \| **Tree** \| **Deprecated** \| **Index** \| **Help** \| **Weka's home** | |  |
| **PREV CLASS**   **NEXT CLASS** | **FRAMES**    **NO FRAMES**     **All Classes** |  |
| SUMMARY: NESTED \| FIELD \| CONSTR \| METHOD | DETAIL: FIELD \| CONSTR \| METHOD |  |


---


## weka.classifiers.lazy Class LBR

```
java.lang.Object
  weka.classifiers.Classifier
      weka.classifiers.lazy.LBR
```

**All Implemented Interfaces:**: java.io.Serializable, java.lang.Cloneable, CapabilitiesHandler, OptionHandler, RevisionHandler, TechnicalInformationHandler

---

``` public class LBR extends Classifier implements TechnicalInformationHandler ```

Lazy Bayesian Rules Classifier. The naive Bayesian classifier provides a simple and effective approach to classifier learning, but its attribute independence assumption is often violated in the real world. Lazy Bayesian Rules selectively relaxes the independence assumption, achieving lower error rates over a range of learning tasks. LBR defers processing to classification time, making it a highly efficient and accurate classification algorithm when small numbers of objects are to be classified.
